# A Response to Commentaries

**DOI:** 10.1002/hast.659

**Published:** 2016-11-21

**Authors:** I. Glenn Cohen, Holly Fernandez Lynch, Christopher R. Deubert

## Abstract

*Our article “NFL Player Health Care: Addressing Club Doctors’ Conflicts of Interests and Promoting Player Trust” focused on an inherent structural conflict that faces club doctors in the National Football League. The conflict stems from club doctors’ dual role of providing medical care to players and providing strategic advice to clubs. We recommended assigning these roles to different individuals, with the medical staff members who are responsible for providing player care being chosen and subject to review and termination by a committee of medical experts selected equally by the NFL and the NFL Players Association. Recognizing that the problem of structural conflict of interest is deeply entrenched and that our recommendation is a significant departure from the status quo, we invited comment from a diverse and highly qualified group of experts. There is considerable common ground among the commentators. All but one agreed with us that, despite the best intentions of upstanding professionals, there is a structural conflict of interest in the club doctors’ relationship with players, and the commentaries were generally supportive of our recommendation for change. There are also meaningful disagreements, however. Some commentators think that the proposal is on the right track but does not go far enough to reduce the structural conflict of interest, and one commentary wholly disagrees with our analysis and recommendations.*

## Response

Our article, “NFL Player Health Care: Addressing Club Doctors’ Conflicts of Interests and Promoting Player Trust,” focused on an inherent structural conflict that faces club doctors in the National Football League.^1^ The conflict stems from club doctors’ dual role of providing medical care to players and providing strategic advice to clubs. We recommended assigning these roles to different individuals, with the medical staff members who are responsible for providing player care being chosen and subject to review and termination by a committee of medical experts selected equally by the NFL and the NFL Players Association. Recognizing that the problem of structural conflict of interest is deeply entrenched and that our recommendation is a significant departure from the status quo, we invited comment from a diverse and highly qualified group of experts.^2^ We thank the commentators for being a part of this process.

There is considerable common ground among the commentators. All but one agreed with us that, despite the best intentions of upstanding professionals, there is a structural conflict of interest in the club doctors’ relationship with players, and the commentaries were generally supportive of our recommendation for change. Marvin Washington perhaps best captures our discussion of the problem, declaring that “[m]any club doctors are good people” but that “the structure of the system [in which they provide care] is not optimal, for the player or the doctor.”^3^ Indeed, Laurent Duvernay‐Tardif, a current NFL player and medical student, stated that, “if the conflicts can be reduced or avoided by making structural changes to medical practice, doing so seems laudable.”^4^

There are also meaningful disagreements, however. Some commentators think that the proposal is on the right track but does not go far enough to reduce the structural conflict of interest, and one commentary wholly disagrees with our analysis and recommendations.

## Limitations of Our Proposal

Our recommended approach falls short of absolute bifurcation between the medical staff members serving players and those serving clubs, based on the realities facing players in need of care and clubs in need of information. Not surprisingly, two sets of commentators put pressure on this approach, and we acknowledge some of its limitations.

Arthur Caplan, Brendan Parent, and Lee Igel affirm that players must be provided a medical staff exclusively devoted to their interests, but they propose a system in which, rather than relying on any medical staff members provided via the employment relationship, players should be required to locate and pay their doctors on their own.^5^ We think our proposal has some practical advantages, however. Under our proposal, players would not have to strike off on their own to receive care and treatment, although they would continue to have a right to seek care outside the club medical structure. We believe relying solely on that existing right and eliminating care available “on the job,” as proposed by Caplan et al., imposes burdensome transactional costs on players and fails to recognize the constantly changing circumstances of their lives. Very few players currently maintain relationships with doctors outside the club, for a variety of logistical reasons. Moreover, even if external doctors were granted access to club facilities and authority to make game‐time decisions, there would be inevitable logistical concerns with implementing such an approach: Would they attend all practices and games? Would they travel to away games? Would telemedicine be sufficiently protective of players?

We also believe that players should not be required to pay for health care that they need because of employment‐related injuries, conditions, or risks and therefore should not have to pay for their own doctors, as Caplan et al. suggest; instead, these costs should fall to the club as the players’ employer. And if the club is paying, there is little reason to prefer a system in which players exclusively retain their own doctors over a system, as we propose, in which players have access to doctors selected and reviewed by an expert committee. Indeed, our approach offers more protection of players, given the system of peer review we recommend.

While Caplan et al. argue that we should go further, Ross McKinney suggests that we may have gone too far, at least as a political matter.^6^ McKinney agrees that our approach *should* be implemented, but worries that it may be too “culturally alien” to the NFL (p. S34). Our recommendation is indeed a substantial deviation from the historical practices of NFL clubs and their medical staffs and will likely require further study and adjustment. We agree that the NFL and its clubs might resist this approach because it would lessen their control over players and their medical care. But the bottom line is that few of us would fully trust a doctor hand‐picked by our employer, serving entirely at the employer's pleasure, and with distinct obligations to the employer. Why should NFL players have to tolerate such a system?

To be sure, our recommendation does not resolve all trust concerns because it still permits player medical information to flow to the club via what we call the “Players’ Medical Staff.” As a result, some players will probably sometimes still withhold information about their conditions to ensure that it is not relayed to the club. We do not believe there is any realistic system that could fully resolve this issue, given the club's business interest in player health.

## Ongoing Debate about the Problem

The only commentator to wholly disagree with our recommendation is the NFL Physicians Society.^7^ Unfortunately, the NFLPS spends very little time discussing the details of our proposal; instead, it argues that the current system presents no real conflict of interest at all. In other words, it rejected our very premise, thereby contradicting an overwhelming body of literature,^8^ including the other commentaries in this special report and the American Medical Association's *Code of Medical Ethics*.^9^

The NFLPS seems to regard our analysis as an unfair attack on highly qualified and ethical club doctors. We take great pains in “NFL Player Health Care” to make clear that this is not what we intend. We are not making a moral judgment about club doctors as competent professionals or devoted individuals. Instead, we are taking issue with a health care structure that requires club doctors to “simultaneously perform two roles that are not necessarily compatible.”^10^ The NFLPS also argues that the conflict of interest we identify is merely “theoretical.”^11^ To see why this is erroneous, consider an analogy to the way in which structural conflicts of interest are avoided in organ donation. Both law and ethics require two separate care teams: one to care for dying patients and pronounce them dead and one to conduct the transplant and care for the recipient.^12^ If a single medical team served both roles, it would face the structural problem of dual loyalty, to the dying patient and to the patient in need of transplant, even though the interests of the two parties may conflict. In the organ transplantation context, this bifurcation of roles is well established and mandatory—even if, for example, an individual doctor would swear that he or she is not influenced in declaring a donor's death by the desire to get his or her patient an organ and even though it would be impossible in any particular case to prove or disprove such influence.

The NFLPS goes on to argue that club doctors are merely “medical messengers.”^13^ This argument is belied by club doctors’ own obligations. Paragraph 8 of the standard NFL player contract provides that it is the exclusive responsibility of the club doctor to determine whether the player has “maintain[ed] his excellent physical condition” and that, if he has not, the club may terminate the player's contract.^14^ The medical information that club doctors provide to clubs is essential to this determination.

The NFLPS tries to excuse the structural conflict by citing the broad confidentiality waivers players execute authorizing the NFL, all NFL clubs, clubs’ medical staffs, and others to use and disclose player health information. The NFLPS's reliance on these waivers is misplaced, however, as players are without meaningful options. There is no doubt that players execute these waivers of their legal rights because they fear that if they do not, their contracts will be terminated. This is a practice that is itself ethically questionable, as pointed out by Mark Rothstein in his commentary,^15^ and one that exacerbates and does not excuse the embedded structural conflict of interest of the current system.

The NFLPS also argues that the recommendations are based on poor research. It takes issue with the methodology and the sample size of players we interviewed, arguing that the sample was insufficient to determine that there is a problem with the current structure of NFL player health care. We agree that the interviews cannot serve that purpose. As we state in the article, these quotations are illustrative—not representative—of players’ views; their purpose is to let players speak in their own voices about a problem amply documented in the existing literature and recognized by the other commentators. We think that, even if we had not engaged in any interviews at all, simply examining the structure of NFL clubs’ medical staffs would be sufficient for our analysis, as the structure itself presents a clear conflict of interest. The NFLPS also criticizes us for not directly engaging club doctors in our research on this issue. Actually, as the NFLPS is aware, we sought to interview club doctors but were unable to gain access to them. In 2014, we notified the NFL that we intended to seek interviews with club personnel, but the NFL advised us that it was “unable to consent to the interviews.”^16^ Without the consent of the NFL, whose members employ the club doctors, we did not believe the interviews could be successful, and we decided not to pursue them. In 2016, when we engaged the NFLPS about providing a commentary for this special report, the NFLPS wanted to know how many club doctors we had interviewed, and we responded that we had not interviewed any but would welcome the opportunity to do so. Indeed, we offered to delay publication to make the interviews possible. The NFLPS declined our invitation.^17^

Moreover, we note that in 2015, the joint NFL‐NFLPA Accountability and Care Committee administered a survey to NFL players about the quality of NFL player health care. If NFLPS believes that there is no support for our contention that players believe that club doctors are conflicted, surely that will be reflected in the survey's results. Therefore, we call on the NFLPS (in cooperation with the NFL and NFLPA, as needed) to publicly release those survey results.

Finally, the NFLPS's discussion of our recommendation appears to misunderstand some of its components. Our recommendation would not change the number of doctors providing care to players in a meaningful way. Clubs currently typically pay for two levels of care: the primary care by the club doctor and then also a second opinion obtained by the player. Our proposed structure does create a potential third layer of medical examination, that of the Club Evaluation Doctor. Nevertheless, we disagree that this is a problem for several reasons: our proposed structure is essential for players to receive minimally conflicted health care; with the addition of a Head Players’ Doctor entirely devoted to the players’ interests, players should have an increased level of trust in their primary level of care, which may decrease the need for and cost of second opinions; clubs would also benefit from our recommended arrangement by having a Club Evaluation Doctor who would be entirely devoted to the club's interests; and, at least under the current collective bargaining agreement, some of the costs of medical care, including physical examination costs, are at least partially paid for out of the players’ share of revenue (in other words, additional costs for player health care can decrease the amount of money available to players in salary).^18^

Additionally, the “game‐day interplay” would not be logistically challenging: an injured player would be treated by a Players’ Doctor and a Players’ Athletic Trainer, who would determine whether the player is able to return to play and would advise the Club Evaluation Doctor accordingly. Our recommendation would not require the Head Players’ Doctor to attend all practices; the Head Players’ Doctor's involvement would mirror the current involvement of club doctors—attending practices sporadically and relying on the Player's Athletic Trainer for the bulk of the players’ treatment.

Moreover, we think that the “reduced level of communication”^19^ under our recommendation is not problematic for player health care but, rather, protective of it. In receiving health care, a player would have complete and unfettered communication with the Players’ Medical Staff providing his treatment, and members of that staff would have unfettered communication with one another. The only reduced communication is that between the Players’ Medical Staff and the club—to protect the integrity of the health care provided to the player.

We hope that the NFLPS will reconsider its stance on these issues and join with the other commentators (and the vast literature) in acknowledging the structural conflict of interest at hand. Neither players, nor clubs, nor club doctors should prefer the status quo, and the NFLPS would be a valuable partner in working toward a better system. We invite and look forward to that further discussion with all interested stakeholders.
